# Understanding the traits underlying vaccine-driven virulence evolution in malaria parasites

**DOI:** 10.1186/s12915-025-02366-w

**Published:** 2025-08-26

**Authors:** Youngseo Jeong, Tsukushi Kamiya, Nicole Mideo

**Affiliations:** 1 Department of Ecology and Evolutionary Biology, University of Toronto, Toronto, Canada; 2 Center for Interdisciplinary Research in Biology (CIRB), College de France, Paris, France

**Keywords:** *Plasmodium chabaudi*, AMA-1, Mathematical modeling, Immune evasion, Erythropoiesis, Anemia, Within-host dynamics

## Abstract

**Background:**

Vaccine-driven evolution can erode the beneficial effects of vaccination and is a concern, especially for newly introduced vaccines. While obvious candidates for vaccine-driven evolution are the precise parasite antigens that are the targets of vaccine-induced immunity, traits underlying parasite virulence may also evolve. Previous experimental work in rodent malaria demonstrated that evolution in vaccinated hosts resulted in increased parasite virulence, as measured by anemia (minimum red blood cell density). However, no genetic changes were detected at vaccine target sites, leaving the underlying traits or their interactions with host responses unclear. Using a hierarchical Bayesian framework, we fitted a mathematical model of within-host malaria infection dynamics to experimental time series data from infections in mice inoculated with parasites that had evolved in either vaccinated mice or sham-vaccinated (control) mice. We compared parameter estimates across treatments to understand which parasite traits could plausibly explain differences in infection dynamics and virulence.

**Results:**

Vaccine-evolved parasites elicited lower targeted immune killing and anemia-driven erythropoiesis, differences that were observed at the level of treatment means and when accounting for individual-level variation. We validated our model by calculating early-infection parasite multiplication rates, finding no differences across treatments (either experimental or simulated)—differences that would be expected if the vaccine target antigen (AMA-1) had evolved.

**Conclusions:**

Our results emphasize the complexity of virulence, showing that parasite modulation of host responses can influence disease severity. We also highlight the important role for evolution of parasite traits beyond target antigens in response to vaccination.

**Supplementary Information:**

The online version contains supplementary material available at 10.1186/s12915-025-02366-w.

## Background

Vaccines, while highly effective at reducing disease burden, can sometimes fail to perfectly prevent infection and transmission, which creates opportunities for parasite evolution [1, 2]. Parasite adaptations against vaccines can be put into two, non-mutually exclusive categories: antigenic evolution and the evolution of parasite life history traits. Antigenic evolution occurs when mutants with antigens that are not recognized by the vaccinated host’s immune system arise and/or increase in frequency. Because this type of adaptation is due to mutations at vaccine target antigens, and the genetics underlying these antigens are often well-known, it can be detected in the genetic sequences [3, 4]. Evidence for vaccine-driven antigenic evolution has been shown for vaccine-preventable human diseases, like pneumococcal infections and pertussis [3, 4] and is one of the contributing factors that necessitate regular updates to the seasonal influenza vaccines.

Alternatively (or in addition), vaccine-induced evolution may alter parasite life history traits. For example, changes to traits like replication rate may confer a selective advantage in a vaccinated host [5]. Unlike antigens, these traits are likely to be the product of multiple interacting genes, making their evolution more difficult to identify. When these traits are related to virulence (i.e., damage caused to hosts or disease severity), their evolution not only erodes the protective effects of vaccines against infections but also causes greater harm for non-vaccinated individuals in the population once infected [1, 6].

Mathematical models have identified certain conditions under which imperfect vaccines (i.e., those that do not completely block infection or transmission) can favor parasites with higher virulence [7]. In cases where vaccines reduce within-host parasite growth, and growth is positively correlated with both transmission and virulence, vaccines indirectly reduce parasite fitness. In such cases, mutants with higher within-host growth can achieve more transmission and greater fitness, while causing less damage or host death than in the absence of vaccines. In other words, if a vaccine effectively reduces the cost of higher virulence, higher intrinsic virulence is favored [7]. Virulence evolution may proceed in the opposite direction if vaccines directly target other processes, like establishing an infection or onward transmission [7], or there may be no detectable evolutionary response if virulence is a consequence of the parasite’s interactions with innate immunity and thus independent of the effect of the vaccine [8]. Finally, the evolutionary outcome is dependent on the extent to which vaccines reduce mortality and/or transmission, and any heterogeneity in vaccine effects across hosts [9].

One experimental example of vaccine-driven virulence evolution comes from a rodent malaria system. *Plasmodium chabaudi* parasites were serially passaged via intraperitoneal syringe injection through naïve (non-vaccinated) mice or mice vaccinated with an apical membrane antigen (AMA-1) [10]. AMA-1 is a candidate blood-stage malaria vaccine antigen that is involved in the invasion of the host’s red blood cells (RBCs) by the parasite’s asexual life stages [11, 12]. *P. chabaudi* parasites replicate inside RBCs, eventually causing them to burst and release progeny parasites; it is this cycle of invasion, replication, and bursting that leads to disease symptoms. As such, vaccination with AMA-1 should lead to immune responses that directly target within-host parasite growth. After 21 passages in vaccinated mice, the evolved parasites were more virulent, causing greater anemia (measured as minimum RBC density during the acute phase of infection in a naïve host) than those passaged through non-vaccinated mice [10]. Total parasite density in the acute phase of infection was also assessed and showed a similar trend, though the difference was not statistically significant. Intriguingly, the researchers found no evidence for evolution at the AMA-1 locus in parasites from either selective treatment group. This suggests that the observed higher virulence was not related to antigenic evolution and instead was due to changes in other parasite traits.

Virulence of malaria parasites is a product of many traits and their interactions with host responses, such as exploiting host resources (RBCs) and interfering with their production [13], interfering with host immune mechanisms [14], cytoadherence [15], and toxin production [16]. Thus, it is unclear what trait (or traits) in particular can explain the observed increase in virulence in parasites that evolved in vaccinated hosts. To elucidate the mechanistic underpinnings of vaccine-driven virulence evolution in rodent malaria parasites, we fit a mathematical model of blood-stage infection to experimental data using a hierarchical Bayesian framework. By comparing parameter estimates between the experimental treatments, we identify the specific parasite traits and interactions with host processes that can plausibly explain the observed evolution of higher virulence in vaccinated hosts. Finally, we calculate predicted parasite multiplication rates (PMRs) in the earliest days of infection when the parasites are not resource-limited. Since functional changes of the AMA-1 protein, or others that interact with it, are expected to influence the parasite’s invasion of RBCs [17], we partially validate our model by finding no difference across treatments in PMRs, consistent with the empirical result of no observed genetic changes in AMA-1 [10].

## Results

### The mechanistic model captures experimental infection data

**Fig. 1:**
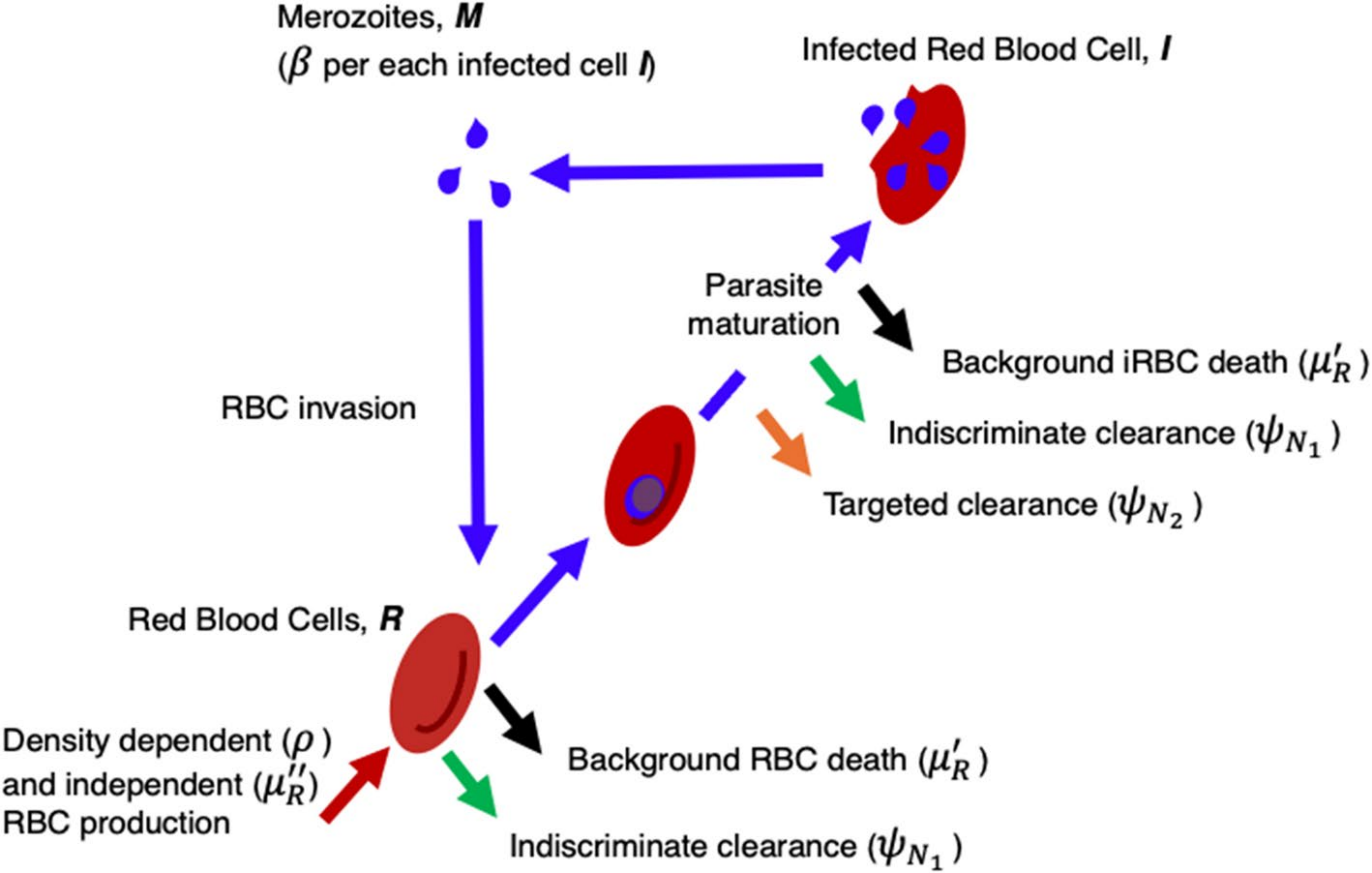
Model schematic, showing the dynamics of RBCs and iRBCs within a host. Each loop following the purple arrows represents the 24-h life cycle of the parasite, corresponding to 1 day ($t=0$to$t=1$) in the model. Indiscriminate immune clearance of all RBCs and targeted immune clearance of iRBCs are shown in green and orange arrows, respectively. Production and background death of RBCs are shown in red and black arrows

We fitted a mechanistic model of blood-stage malaria infection and host responses, developed by Kamiya et al. [18], to experimental time series of infections in naïve mice inoculated with parasites that had evolved in either vaccinated or sham-vaccinated mouse hosts [10]. Briefly, the model tracks RBC and infected RBC (iRBC) densities in discrete time and includes different components of host RBC production and immunity (Fig. 1). Specifically, we included both density-dependent and density-independent forms of RBC production to capture compensatory RBC production in response to anemia [13] and background production of RBCs maintaining homeostasis, respectively. Our model phenomenologically captures killing of iRBCs and, separately, all RBCs, corresponding to ‘targeted’ and ‘indiscriminate’ immune responses that are both assumed to be activated by the density of iRBCs [19]. The experimental data from Barclay et al. [10] includes RBC density measured on the day of inoculation (day 0) and then daily RBC and iRBC density measures from days 3 to 20 post-inoculation for 25 individual mice inoculated with 10^6^ parasites from either the vaccine-evolved (*n* = 13) or sham-vaccine-evolved (*n* = 12) lines. We focus our analysis on data from days 0 to 10, which encompasses the first wave of infection. We fitted the model parameters to the data using a hierarchical Bayesian framework with both treatment- and individual-level effects.

**Fig. 2:**
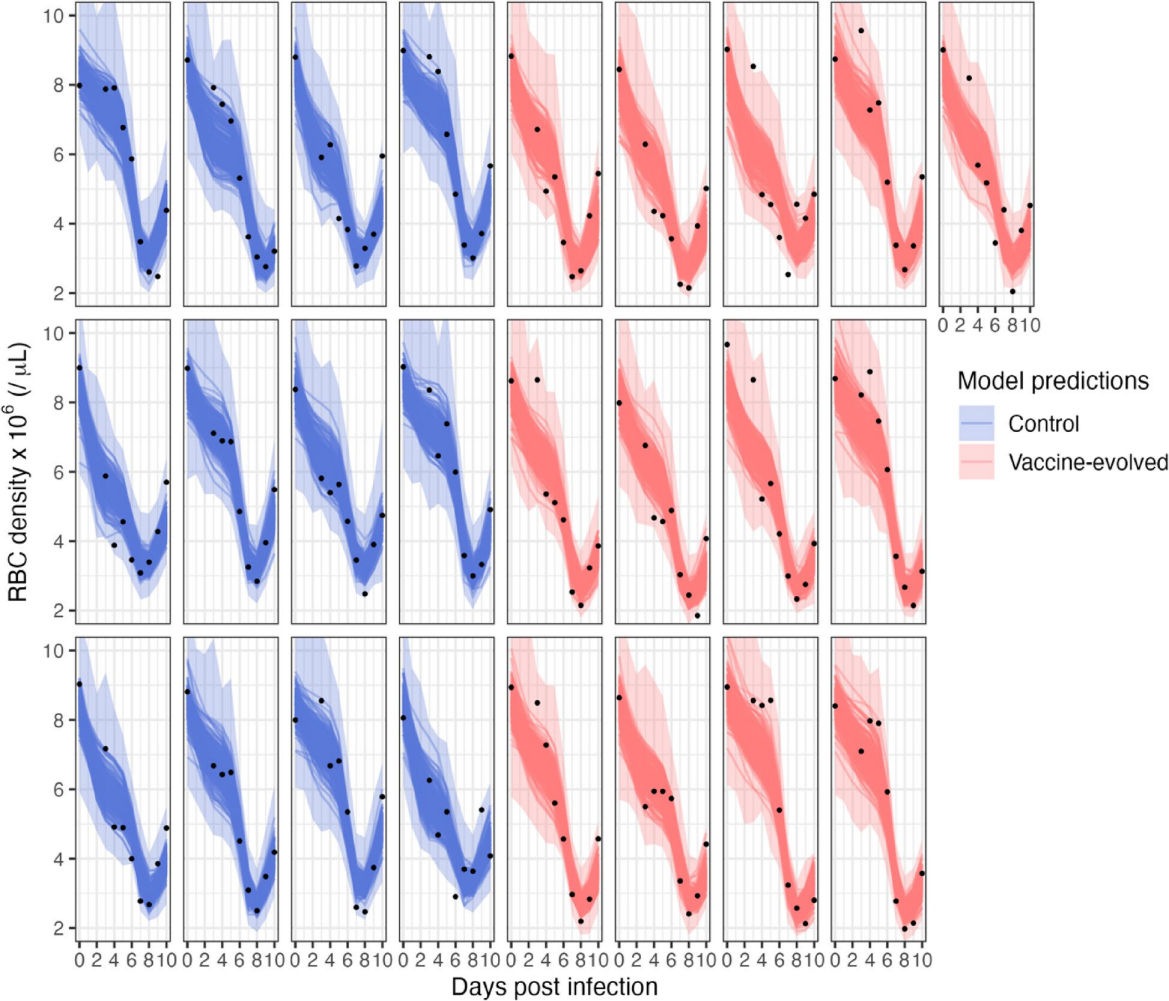
Fit of the dynamic model to RBC time series data (black points) for each individual infection. The dark lines are model predicted dynamics from 200 parameter sets chosen from the posterior distribution and the light-colored bands are the 95% posterior predictive intervals, which incorporate uncertainty in parameter estimation and measurement error. To incorporate measurement error, we took the predicted dynamics (the dark lines) and took random draws from the log-normally distributed error structure and the estimated standard deviation of measurement error (${\sigma }_{RBC}$), then calculated the 95% intervals

**Fig. 3:**
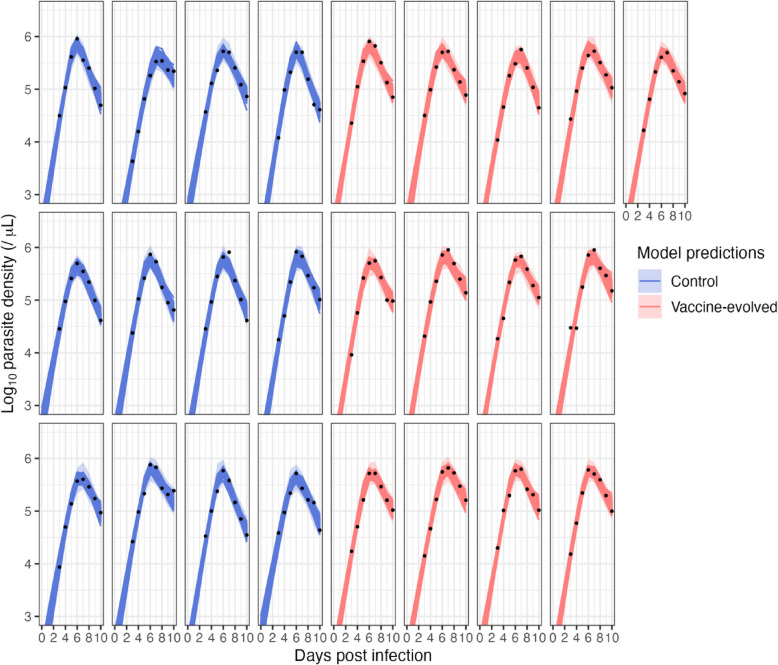
Fit of the dynamical model to iRBC time series data (black points) for each individual infection. As with Fig. 2, the dark lines are the model predicted dynamics from 200 posterior samples*,* and the light-colored bands are the 95% posterior predictive intervals with measurement errors. The individual model predictions overlap the 95% posterior predictive intervals because the estimated standard deviation of measurement error for log parasite density is small (0.1/µL)

Figures 2 and 3 illustrate that the fitted model adequately captures the dynamics of RBCs and iRBCs and provides a good fit to the data with no sign of bias (Additional file 1: Fig. S1). Posterior predictive checks demonstrate that parameter estimation was sufficiently informed by the data while not overfitting to the data (Additional file 1: Fig. S2) [20]. Estimation of initial RBC and iRBC densities is strongly informed by the priors, but that is expected since we used specific, informative priors taken from the real data (RBC) or our expectation of the data (iRBC; see Methods for more details). Pairwise parameter collinearity, measured as correlation coefficients, is below 0.6 for all fixed effects (Additional file 1: Fig. S3), indicating no issues with practical parameter identifiability.

### Characterizing the differences between treatments

**Fig. 4:**
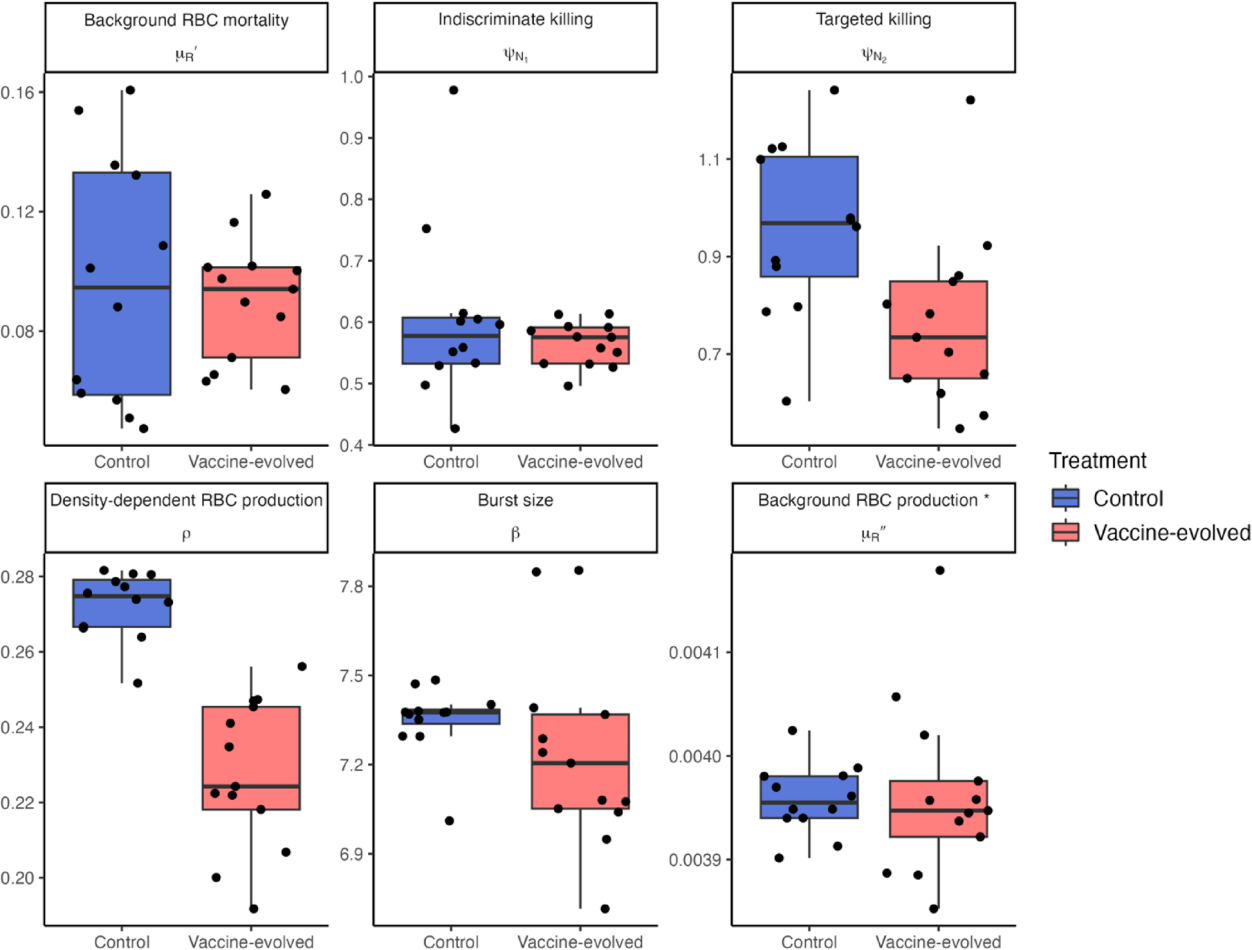
Box plots of individual-level median estimates for each fitted parameter, indicated by the panel label. Each point represents a median estimate for an individual mouse. The asterisk indicates that the mean background RBC production was fixed to a previously estimated value (Table 1); hence, only individual-level variation was fitted and not treatment-level effects

**Fig. 5:**
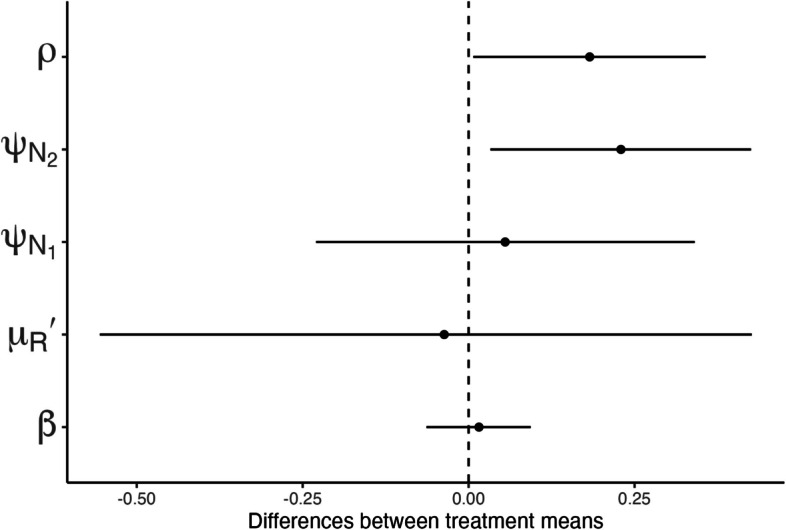
Forest plot of differences between the estimated treatment means. The horizontal lines indicate 95% credible intervals*,* and the points indicate the means. Deviations of the lines from being centered at zero indicate a difference between the treatments

Median parameter estimates for individual mice suggest some differences between the parasites that evolved in vaccinated versus non-vaccinated hosts (Fig. 4). For more quantitative analyses, we characterized the differences in parasite traits and their interaction with host processes at two levels, focusing on treatment means or individual mice, mirroring the hierarchical organization of our parameter estimation approach. First, at the treatment level, we calculated the predicted differences in treatment means for each parameter and assessed whether the 95% high density interval deviates from zero (Fig. 5). We found that both the activation rate of targeted immune killing, ${\psi }_{{N}_{2}}$, and the density-dependent rate of RBC production, $\rho$, are significantly lower in infections with vaccine-evolved parasites. We note that burst size and merozoite invasion rate could not be estimated simultaneously due to identifiability issues, and we report results from fitting burst size and fixing invasion rate. Fitting invasion rate and fixing burst size produced qualitatively similar results (Additional file 1: Fig. S4).

**Fig. 6:**
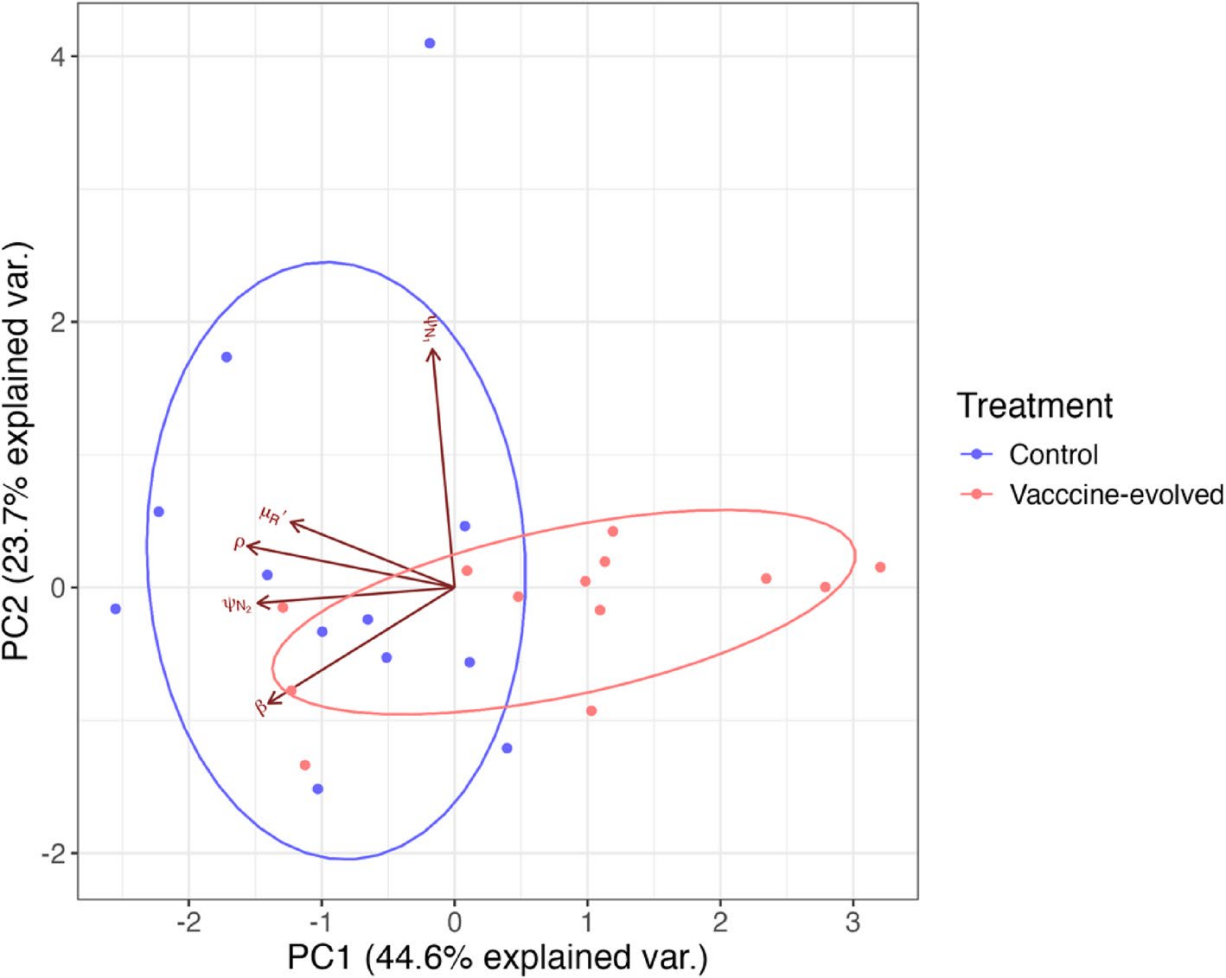
PCA biplot showing the relationship between individual mice in the first two principal components (cumulatively accounting for 64.9% of the variation in individual-level median parameter estimates). The plot reveals the separation between the treatments along PC1. The direction and length of the red arrows indicate  the contributions of each parameter to the principal components. Parameters are described in Table 1 and their individual correlations with each PC axis are reported in Table S1 corr21_3734fa8e-84ee-41eb-b2d1-434426ca6976

Second, we compared individual median parameter estimates in the full, multi-dimensional parameter space using principal components analysis. The logic of this approach is, firstly, to validate the differences that the pairwise comparison has already revealed, since highly varied individual effects could obscure the realized differences in treatment effects for individual mice. Second, by employing a PCA, we can identify patterns in the multi-dimensional parameter space beyond pairwise comparisons. Accounting for variation at the individual-level, the same pattern of lower ${\psi }_{{N}_{2}}$ and $\rho$ in the vaccine-evolved parasites is seen in the multivariate parameter space and there is clear separation between the two treatments along PC1 (Fig. 6; correlations between each parameter and the first two PC axes are reported in Additional file 1: Table S1). The biplot also suggests that the vaccine-evolved parasites tend to induce less background RBC death ($\mu'_{R}$) and have smaller burst sizes ($\beta$) than the control parasites; however, there is little variation in estimates of $\beta$ overall (note y-axis scale in Fig. 4), and the variation captured by PC1 is not representative of the wide range of $\mu'_{R}$ in the control treatment (note the range of $\mu'_{R}$ in the control vs. vaccine-evolved Fig. 4); thus, these parameters are unlikely to be driving the observed dynamical differences.

### Model predictions align with empirical result of no antigenic evolution

**Fig. 7:**
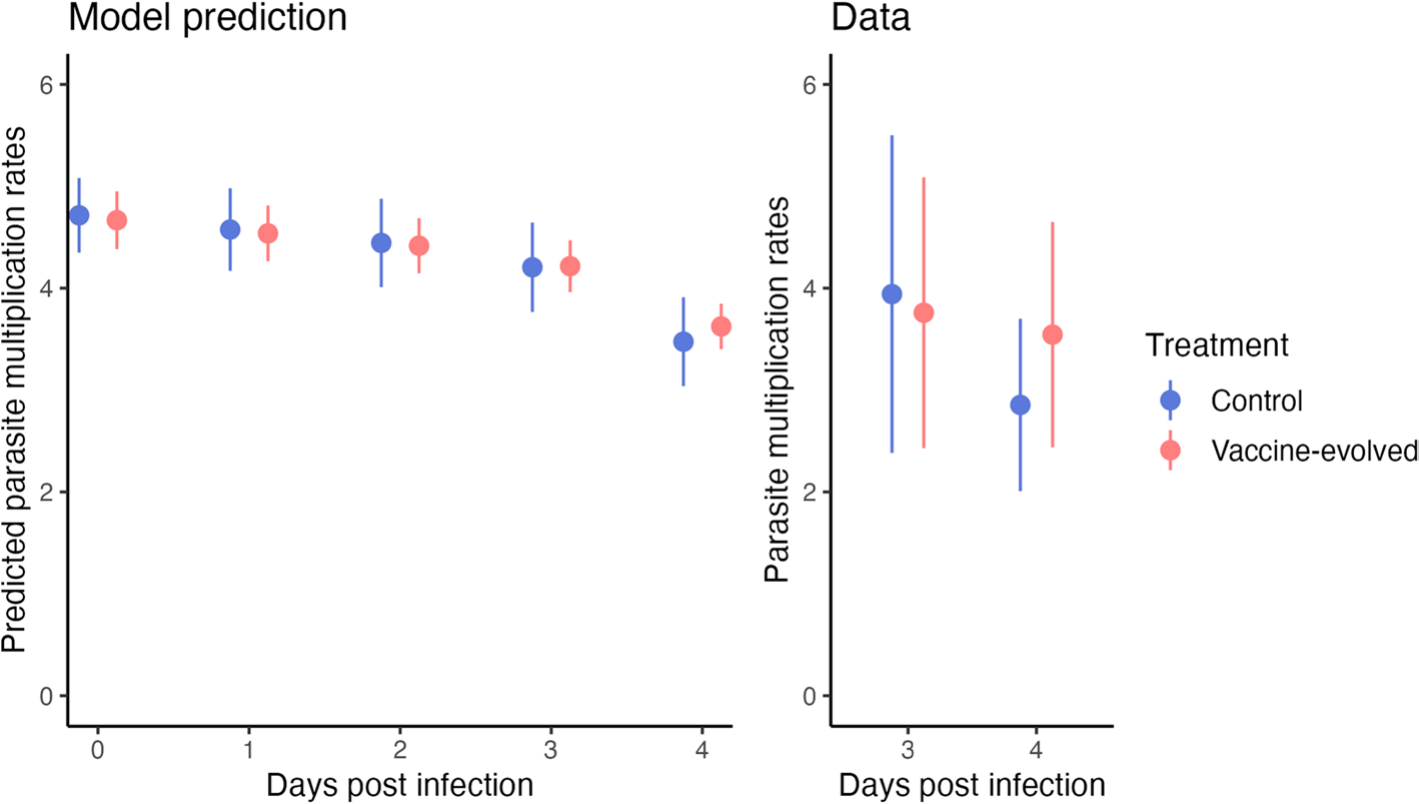
Model predicted parasite multiplication rates (PMRs; left panel) and PMRs calculated from the data (right panel) show similar patterns and no differences between treatments. PMRs are calculated for individual mice as iRBC density on day t + 1 divided by iRBC density on day t. For each mouse, we used 200 sampled posterior predictions with measurement error, as with Fig. 3. Points represent means across individuals*,* and error bars represent standard deviations

In vaccine experiments, the functioning of AMA-1 is often assessed in vitro by comparing parasite growth rate over one replicative cycle [12, 22] since AMA-1 plays a crucial role in asexual multiplication of the parasite by forming a junction with the host RBC during the invasion process [17]. One way of estimating growth rate is through measures of the parasite multiplication rate (PMR), calculated as iRBC density on day $t+1$ divided by iRBC density on day $t$, for a parasite with a synchronous, 24-h replication cycle like *P. chabaudi*. Since Barclay et al. [10] did not detect changes in the epitope sequences of AMA-1, and if other invasion-related parasite proteins did not change, we expect no difference in the function of AMA-1 between the treatments and no differences in PMRs estimated in the earliest days of infection before host immune responses ramp up or resources become limiting. Focusing on the first five days of infection, we find no difference in PMR (calculated from 200 posterior samples, a sample size chosen to balance computational ease and informativeness) across treatments in the experimental data (Fig. 7, right panel; note that the data only allow PMR to be calculated starting on day 3 post-inoculation). From our model predicted dynamics, we calculated PMRs starting at *t* = 0 and confirmed that there are no differences between treatments in the first five days (Fig. 7, left panel). Since iRBC dynamics were not fit to any data prior to day 3 (since those data were not available), this analysis offers a partially independent validation of the model and is consistent with no functional changes AMA-1, though we note that other proteins contribute to the invasion process [23].

## Discussion

In this study, we fitted a mechanistic model of within-host rodent malaria infection dynamics to experimental data to understand which parasite traits and/or their modulation of host responses can plausibly explain the evolution of increased virulence in response to vaccines. In the original experiment, the researchers observed that vaccine-evolved parasites were more virulent, causing greater anemia in naïve hosts, but which parasite traits had evolved remained a mystery since it was not the vaccine-targeted antigen [10]. Our results show that compared to parasites evolved in non-vaccinated hosts, vaccine-evolved parasites elicited a weaker activation of both the targeted immune response (${\psi }_{{N}_{2}})$ and density-dependent RBC production ($\rho )$, leading to more severe anemia in hosts.

Density-dependent RBC production, $\rho$, is a composite parameter in our model since it captures both host responses and the parasite’s modulation thereof. Anemia follows *Plasmodium* infection because successful invasion of an RBC by a parasite results in the destruction of the host cell. Hypoxia caused by this anemia triggers a chain of signaling events leading to compensatory production of RBCs [13]. The activity of this density-dependent production process is captured by our model parameter $\rho$, and a lower value of this parameter in infections with the vaccine-evolved parasites indicates that, for a given deviation in RBC density from the homeostatic equilibrium, hosts are producing fewer new RBCs. One possible mechanistic explanation of this effect is downregulation by hemozoin, a metabolic waste product of digesting hemoglobin by *Plasmodium* parasites. Hemozoin is released along with merozoites when infected RBCs rupture and has been shown to downregulate erythropoiesis in infections across many *Plasmodium* and host species pairs [13, 24, 25]. Hemozoin levels may be higher in the vaccine-evolved treatment due to the total parasite density being higher in these infections, though not significantly so in the experimental data (in our model, the average peak parasite density was 5.91 × 10^6^ and 6.44 × 10^6^ per microliter in the control and vaccine-evolved treatments, respectively). An alternative possibility is that the vaccine-evolved parasites metabolize more or faster, though we might expect that to translate to higher burst sizes ($\beta$), which we do not infer from our model. In any case, this mechanistic explanation could be tested by measuring hemozoin in plasma of hosts infected with the vaccine-evolved versus control parasites, samples that would necessitate new experiments.

The inferred reduction in the activation strength of targeted immunity, ${\psi }_{{N}_{2}}$, in infections with vaccine-evolved parasites compared to control parasites can be interpreted in two ways. First, it might reflect lower immune detection of iRBCs by the innate immune system or, second, it might represent lower immune signaling once those infected cells are detected. These two possibilities are difficult to distinguish because our model does not consider the dynamics of specific immune cells or molecules explicitly, rather, it captures outcomes: immune killing and pathogen clearance [26]. We remind the reader that the experimental data is from infections in naïve, non-vaccinated mice; thus, we expect the innate immune system to be the main contributor to control in these first 11 days of infection. However, the parasites that evolved in vaccinated hosts would have been confronted with an antibody-led immune response that can inhibit parasite replication by targeting the invasion process [27]. Since the antibody target sites did not evolve [10], other mechanisms that could have rescued parasite replication in a vaccinated host include reduced immune signaling due to hemozoin suppressing the activity of dendritic cells [28, 29] or lower immune detection of infected cells via evolution of minor antigens. Interestingly, Bull and Antia [8] recently suggested that higher virulence of vaccine-evolved parasites may not be observed when those parasites infect non-vaccinated hosts, since different components of immunity would be operating and modulating virulence. Our results are counter to this optimism, suggesting that even when the vaccine targets one particular antigen, evolution may occur at non-target sites that interact with innate, rather than adaptive, immunity and result in higher virulence.

Existing polymorphism in AMA-1 in circulating parasites presents a challenge for using this antigen in blood-stage vaccines against human malaria *P. falciparum* [27, 30–33], so the evolution of polymorphism—or lack thereof—in this locus is of particular interest. Barclay et al. [10] sequenced the AMA-1 epitope of evolved parasites, finding no genetic differences from ancestral parasites, but it is possible that changes in other parts of the AMA-1 gene or other genes affected the expression of AMA-1. Our model results are not consistent with this possibility and reaffirm that AMA-1 function did not change during experimental evolution. AMA-1 plays an important role in merozoite invasion of uninfected RBCs, so if there is some meaningful, functional difference in AMA-1 in the vaccine-evolved lines, we would expect the parasite multiplication rates to be different in the earliest days of infection. Estimates of PMR—either from the data or model dynamics—represent an aggregate measure of all parasites within an infection, rather than a measure of individual parasite phenotypes. While undetected polymorphic variants may have been present at low enough frequency to not be sampled (less than 20%) [10], the lack of difference in PMRs between vaccine-evolved and control parasites suggests that any such variant was either too rare to influence the aggregate measure or was not functionally different from the dominant variant.

Balancing selection, driven by acquired immunity in hosts, has been found to contribute to the maintenance of AMA-1 polymorphism in nature [11, 30, 31], and there is robust evidence for the role of such selection in maintaining polymorphisms in other antigens [31, 34]. Adaptive immunity is primed against specific parasite antigen variants, leaving it vulnerable to other variants. If hosts have different infection histories (i.e., have harbored infections with different antigenic variants), then that host population presents a mosaic of selection pressures, each individually favoring different antigenic variants and maintaining polymorphism in the parasite population (e.g., [35]). Unlike in nature, the hosts in the Barclay et al. [10] experiment presented a more homogeneous selective environment for the parasites: every mouse was the same age, sex, and inbred strain, with no prior infection. Thus, the relatively low variation in the selection environment (outside of the focal influence of vaccination) over the course of 21 passages and 147 parasite generations in this experiment may have contributed to the lack of new AMA-1 variants and should not be viewed as robust evidence for low risk of de novo antigenic evolution in response to vaccines for human malaria.

At the same time, circulation in nature also presents a number of constraints on parasite evolution that did not exist in the experimental protocol. In particular, the complex formed by AMA-1 and interacting proteins plays a role in the invasion by parasites of other tissues, such as mosquito salivary glands and mammalian hepatocytes [36]. In the natural parasite life cycle, any functional changes in AMA-1 could pleiotropically affect important host-invasion processes by other life stages, further constraining adaptive evolution against recognition by the vaccine-induced antibodies. The experimental protocol in Barclay et al. [10], as in most rodent malaria experiments, bypassed the liver stage of infection and parasites never experienced selection in a mosquito vector. We therefore suggest that the most evolutionary labile traits, in response to vaccination, are those whose expression is limited to the specific, relevant life stage (e.g., the blood stage processes identified above), or are otherwise unlikely to be exposed to conflicting selection pressures in different host environments.

## Conclusions

Our results reveal the mechanisms that may underlie the previously observed, but not yet understood, vaccine-driven evolution of increased virulence in rodent malaria parasites. We show that vaccine-evolved parasites cause more severe anemia in a naïve host by modulating the host’s erythropoiesis and immune killing response. Compared to infections with parasites evolved in non-vaccinated mice, infections with vaccine-evolved parasites exhibit lower targeted immune killing and anemia-driven RBC production. With interest in the development of AMA-1 vaccines that confer strain-transcending immunity [22], understanding which parasite traits, other than polymorphism in the AMA-1 locus, can evolve in response to vaccine-primed immunity is important. Our results provide insight into vaccine-driven parasite evolution and its consequences for virulence—a product of complex interactions between parasites and hosts*—*and emphasize the potential for changes in off-target traits to erode the benefits of vaccination.

## Methods

### Experimental data

Barclay et al. [10] conducted an experimental evolution study, passaging rodent malaria parasites (*Plasmodium chabaudi adami* DK strain) through mice either vaccinated with AMA-1 protein (from *P. c. adami* DK strain, mixed with Montanide ISA adjuvant and PBS) or with a “sham” vaccine (adjuvant and PBS; below we refer to the parasites evolved in this latter treatment as “control”). Evolved parasites were assessed in a variety of host environments. We focus our attention on “evaluation experiment 3”, where naïve (i.e., non-vaccinated) C57Bl/6 mice were inoculated with 10^6^ vaccine-evolved (*n* = 15) or control parasites (*n* = 14). All infections were initiated with intraperitoneal injection of blood-stage parasites. The experimental dataset includes near-daily measures of RBC and iRBC densities from days 0 to 20 post-inoculation. The full experimental methods can be found in Barclay et al. [10]. Because virulence was assessed experimentally at day 8 or 9 (peak of anemia) and because our mathematical model does not capture adaptive immune activity that would be active later in infections, we fitted the model to data from days 0 to 10 only, i.e., the first wave of infection. We also excluded data from four mice (two from the control and two from the vaccine-evolved treatment) in our analyses because their infection progression was much slower than the others. This pattern is characteristic of an initial parasite dose that is ordersofmagnitude lower, which has been shown to affect estimated model parameters [37].

### Mechanistic model of within-host infection dynamics

We fitted the experimental data to a previously published mechanistic model of the replication cycle of blood stage *P. chabaudi* infection [18], which tracks the densities of uninfected RBCs ($R_{(d, t)}$) and infected RBCs ($I_{(d, t)}$) in discrete, 24-h cycles. For clarity of explanation, time ($t$) within a day ($d$) is represented as the interval from 0 to 1, where 0 is immediately after midnight of the previous day ($d-1$), and 1 is midnight of a given day ($d$). Immune action is assumed to take place throughout the day ($t=0$ to $t=1$), while the turnover of RBCs, bursting, and parasite invasion of RBCs happens instantaneously at the end of the day, at time $t=1$, given the synchronous bursting of infected cells and short lifespan of extracellular parasites.

Immune action is represented as a rate of RBC killing, and we include two responses that target either all RBCs indiscriminately (${N}_{1}$) or only infected RBCs (${N}_{2}$). Both processes are assumed to be activated in proportion to the density of iRBCs, consistent with how recognition of iRBCs by the innate immune response triggers the series of events that lead to RBC and iRBC killing [19]. Bystander killing of uninfected RBCs (i.e., ${N}_{1}$) has been shown to be important in modeling the decline in the first wave of parasitemia [38], and empirically has been suggested to result from immune responses killing uninfected RBCs that have been altered by parasite proteins [39, 40]. Immune killing rates are determined by the number of infected cells in the system at the beginning of the day ($t=0$), as a proportion of the maximum iRBC density observed in the dataset, and the respective activation strengths (${\psi }_{{N}_{1}}$ and ${\psi }_{{N}_{2}}$). As in Kamiya et al. [18], we assume that during the initial wave of infection, the rate of indiscriminate clearance of RBCs (${N}_{1}$) resets every day, while that of the targeted killing (${N}_{2}$) accumulates throughout the course of the infection, as was estimated previously [37]. Thus, the immune killing rates are given by

1 $$N_{1(d,t=1)}={\psi}_{N_1}\times\frac{I_{(d,t=0)}}{I_{max}}$$

2 $$N_{2\left(d,t=1\right)}=\psi_{N_2}\times\frac{I_{\left(d,t=0\right)}}{I_{\mathrm{max}}}+N_{2\left(d,t=0\right).}$$

At the end of each day, uninfected RBCs (${R}_{\left(d,t=1\right)})$ die at rate$N_{1\left(d,t=1\right)}$ due to indiscriminate immune clearance. Anemia results in the release of RBC production cues (erythropoietin) in the blood, which triggers the production of new RBCs: we assume a proportion, $\rho$, of the deviation from the homeostatic equilibrium RBC density (${R}_{c}$) is produced with a time lag of 2 days to account for the time it takes for RBC progenitors to become RBCs [13, 41]. RBCs die by background mortality at rate $\mu^{\prime}_R$, and a constant number of RBCs (${R}_{c}\left(1-{e}^{{\mu }_{R}^{\prime \prime}}\right)$) are replenished. In the absence of infection, $\mu^{\prime}_R$ and ${\mu }_{R}^{{\prime}{\prime}}$ maintain the homeostatic RBC density (i.e., ${\mu}^{\prime}_{R}={\mu }_{R}^{{\prime\prime}}$), but we allow them to differ to account for additional RBC loss due to blood sampling and RBC death that may be independent of RBC dynamics during infection [42]. In summary, the dynamics of RBCs are given by

3 $$R_{\left(d,t=1\right)}=R_{\left(d,t=0\right)\;}\;e^{-\left(\mu^\prime_R+N_{1\left(d,t=1\right)}\right)\;}+R_c\left(1-e^{\mu^{\prime\prime}_{R}}\right)+\rho\left(R_c-\left(R_{\left(d-2,t=1\right)\;}+I_{\left(d-2,t=1\right)}\right)\right),$$

where the first term captures the loss of uninfected RBCs due to natural death or indiscriminate immune killing, the second term captures density-independent RBC production, and the third term describes density-dependent RBC production.

Infected RBCs (iRBCs) are subject to the same indiscriminate immune killing and background mortality as uninfected RBCs, but they experience additional targeted immune killing at rate ${N}_{2\left(d,t=1\right)}$,

4 $$I_{\left(d,t=1\right)}=I_{\left(d,t=0\right)}\;e^{-\left(\mu'_R+N_{1\left(d,t=1\right)}\;+N_{2\left(d,t=1\right)}\right).}$$

At midnight ($t=1$), all surviving iRBCs burst and release $\beta$ merozoites, producing a total merozoite density, ${M}_{\left(d,t=1\right)}=\beta {I}_{(d,t=1)}$. Merozoites either die with morality rate ${\mu }_{M}$, or invade an uninfected RBC at a per capita rate $p$, giving the probability that a merozoite invades an RBC as $\frac{p{R}_{(d,t=1)}}{p{R}_{(d,t=1)}+ \mu_{M}}$. The expected number of invading merozoites per uninfected RBC is then

5 $$\lambda=\frac{M_{\left(d,t=1\right)}}{R_{\left(d,t=1\right)}+{\displaystyle\frac{\mu_M}p}}.$$

Assuming that the probability of successful invasion by a merozoite is Poisson-distributed with the expected number $\lambda$, the probability that a given uninfected RBC is invaded by a single merozoite is $\lambda {e}^{-\lambda }$ and the probability that an uninfected RBC escapes invasion is ${e}^{-\lambda }$. The model assumes that only a single merozoite can successfully invade an RBC, such that multiple invasions result in immediate death of the RBC. The post-invasion densities (denoted with an asterisk) of RBCs and iRBCs are thus given by

6 $$R^*_{\left(d,t=1\right)}=R_{\left(d,t=1\right)}\;e^{-\lambda}$$

7 $$I_{\left(d,t=1\right)\;}^\ast=R_{\left(d,t=1\right)}\;\lambda e^{-\lambda}.$$

$R_{\left(d,t=1\right)}^\ast$and ${I}_{\left(d,t=1\right)}^{*}$ become the RBC and infected RBC densities at the beginning of the following day, i.e., ${R}_{\left(d+1,t=0\right)}$ and ${I}_{\left(d+1,t=0\right)}$.

### Hierarchical Bayesian inference

The model was fitted to RBC and iRBC data using a hierarchical Bayesian framework. For each of the five parameters that the parasite could plausibly affect ($\mu'_{R},{ \psi }_{N1}, {\psi }_{N2}, \rho , \beta$), we estimated a group mean separately for the two treatments, since the parasites from each treatment evolved independently, and we estimated individual-level variation as random effects. The mean density-independent background RBC production, ${\mu }_{R}^{{\prime}{\prime}}$, was fixed to the estimated value of the corresponding parasite strain from Kamiya et al. [18] for both treatments since our preliminary analysis revealed that the data was not informative for this parameter [20]. We fixed the invasion rate, $p$, as in [18] since the invasion rate and burst size cannot be simultaneously estimated in this model (i.e., they are non-identifiable). However, we verified that the key results do not change when we fix burst size ($\beta$) and fit the invasion rate (Additional file 4: Fig. S4), as expected. Furthermore, the initial densities of RBCs and iRBCs at day 0 were estimated because they were either not available (${I}_{\left(0,t=0\right)}$) or there was uncertainty due to measurement error (${R}_{(0,t=0)}$). The priors for ${R}_{(0,t=0)}$ were informed by the mean and the standard deviation of RBC density data at day 0, and the priors for ${I}_{(0,t=0)}$ were informed by a log-linear regression of parasite densities from the initial growth phase (days 3 to 5), extrapolated to day 0. For these initial densities, we fit one global mean for all individuals and individual-level random effects. We estimated the standard deviations for the RBC and iRBC counts with informative priors from Mideo et al. [43]. All priors are listed in Table 1.

**Table 1:** Table of fixed or estimated parameters. We chose weakly informative prior distributions for the parameters and hyperparameters that represent parasite traits and host responses, and otherwise we used informative priors

Symbol	Description	Fixed value or prior	Source
Within-host infection dynamics			
$\mu'_{R}$	Background RBC mortality rate	$0.025\times \text{exp}(\text{StudentT}\left(\text{1,0},2.5\right))$	[44]
${\psi }_{N1}$	Activation strength of indiscriminate immune killing	$\text{exp}(\text{StudentT}\left(\text{1,0},2.5\right))$	
${\psi }_{N2}$	Activation strength of targeted immune killing	$\text{exp}(\text{StudentT}\left(\text{1,0},2.5\right))$	
$\rho$	Density-dependent RBC production rate	$0.25\times \text{exp}(\text{StudentT}\left(\text{1,0},2.5\right))$	[44]
$\beta$	Parasite burst size	$7\times \text{exp}(\text{StudentT}\left(\text{1,0},2.5\right))$	[44]
${\mu }_{R}^{{\prime}{\prime}}$	Density-independent background RBC production rate	$0.0039$	[18]
${R}_{c}$	RBC density at homeostatic equilibrium	${R}_{(0,t=0)}$(see below)	From data
${I}_{max}$	Maximum iRBC density	$906165$	From data
${\mu }_{M}$	Merozoite mortality rate	$48$	[45]
Initial values			
${R}_{(0,t=0)}$	Initial RBC density	$8708200\times \text{exp}(N\left(\text{0,1}\right))$	From data
${I}_{(0,t=0)}$	Initial RBC density	$737\times \text{exp}(N\left(\text{0,1}\right))$	Inferred from data
Hyperpriors			
${\sigma }_{t}$	Standard deviation of treatment-level variation	$\text{exp}(N\left(\text{0,1}\right))$	
${\sigma }_{m}$	Standard deviation of individual-level variation	$\text{exp}(N\left(\text{0,1}\right))$	
Measurement errors			
${\sigma }_{RBC}$	Standard deviation of log_10_ RBC density	$0.034\times \text{exp}(N\left(\text{0,1}\right))$	[43]
${\sigma }_{iRBC}$	Standard deviation of log_10_ iRBC density	$0.2\times \text{exp}(N\left(\text{0,1}\right))$	[43]

Assuming that the measurement errors of RBCs and iRBCs are log-normally distributed [43], then the log-likelihood function for the Bayesian inference process is given by

8 $$\ln L = \sum_{i}^{n_{mice}} \Bigg\{ \sum_{t}^{n_{time}} \ln \bigg\{ \frac{1}{\sigma_{RBC} \sqrt{2 \pi}} exp \bigg[- \frac{D^{RBC}_{i,t} - M^{RBC}_{i,t}}{2 (\sigma_{RBC})^2} \bigg] \bigg\} +\sum_{t}^{n_{time}} \ln \bigg \{ \frac{1}{\sigma_{iRBC} \sqrt{2 \pi}} exp \bigg [- \frac{D^{iRBC}_{i,t} - M^{iRBC}_{i,t}}{2 (\sigma_{iRBC})^2} \bigg ] \bigg\}\Bigg\}$$

where ${D}_{i,t}$ is the log_10_ of the measured RBC or iRBC density for individual *i* at time *t*, and ${M}_{i,t}$ is the log_10_ of the simulated density of RBCs or iRBCs. We fitted the measurement errors (${\sigma }_{RBC}$ and ${\sigma }_{iRBC}$) with informative priors from Mideo et al. [43] (see Table 1). We note that although the assumption of log-normally distributed errors for RBCs is consistent with Mideo et al. [43], it is different than what is used in Kamiya et al. [18]. We make this choice because log-normally distributed errors weigh deviations at low RBC densities more heavily, which is important for accurately predicting our metric of virulence (i.e., minimum RBC density).

The posterior distribution was sampled by running our model in Stan (version 2.32.2), with the MCMC sampling algorithm provided by Rstan interface for R (version 4.2.2). Four parallel independent chains were run, each with 2000 samples and 1000 warmup. We ensured convergence of the chains by confirming that $\widehat{R}=1$ and effective sample size was greater than 500 per chain for all parameters.

The model fits were evaluated by calculating the standardized residuals and comparing them to Bonferroni-corrected 95% confidence intervals following Miller et al. [44] (see Additional file 1: Fig. S1). To assess the accuracy and precision of the posterior distributions and to confirm that the model parameters are sufficiently informed by the data, we calculated the posterior z-score and contraction [20]. We calculated pairwise parameter correlation coefficients between treatment-level mean parameter estimates (200 samples from the posterior) to check that their independent effects on infection dynamics can be distinguished by the likelihood function (Additional file 1: Fig. S3). High collinearity (absolute value of correlation coefficient above 0.7) indicates issues with practical parameter identifiability [21].

## Supplementary Information

Additional file 1: Fig. S1. Assessment of model fit—standardized residuals. Fig. S2. Assessment of posterior accuracy and precision. Fig. S3. Assessment of Posterior correlations. Fig. S4. Differences between the treatment means when invasion rate is fitted. Table S1. Alignment of the model parameters with the principal component axes

Additional file 2. Dataset and R and Stan code used for analysis and visualization

## Data Availability

The dataset analyzed in this article, produced by Barclay et al. [10], and the code for analysis and visualization are included in the additional files.
