# Supplementary material for: Understanding the traits underlying vaccine-driven virulence evolution in malaria parasites
Source: BMC Biol. 2025 Aug 26;23:267. doi: 10.1186/s12915-025-02366-w (PMC12382291; doi:10.1186/s12915-025-02366-w)
Supplement: Supplementary file 1 — Additional file 1: Fig. S1. Assessment of model fit—standardized residuals. Fig. S2. Assessment of posterior accuracy and precision. Fig. S3. Assessment of Posterior correlations. Fig. S4. Differences between the treatment means when invasion rate is fitted. Table S1. Alignment of the model parameters with the principal component axes [file 12915_2025_2366_MOESM1_ESM.docx]

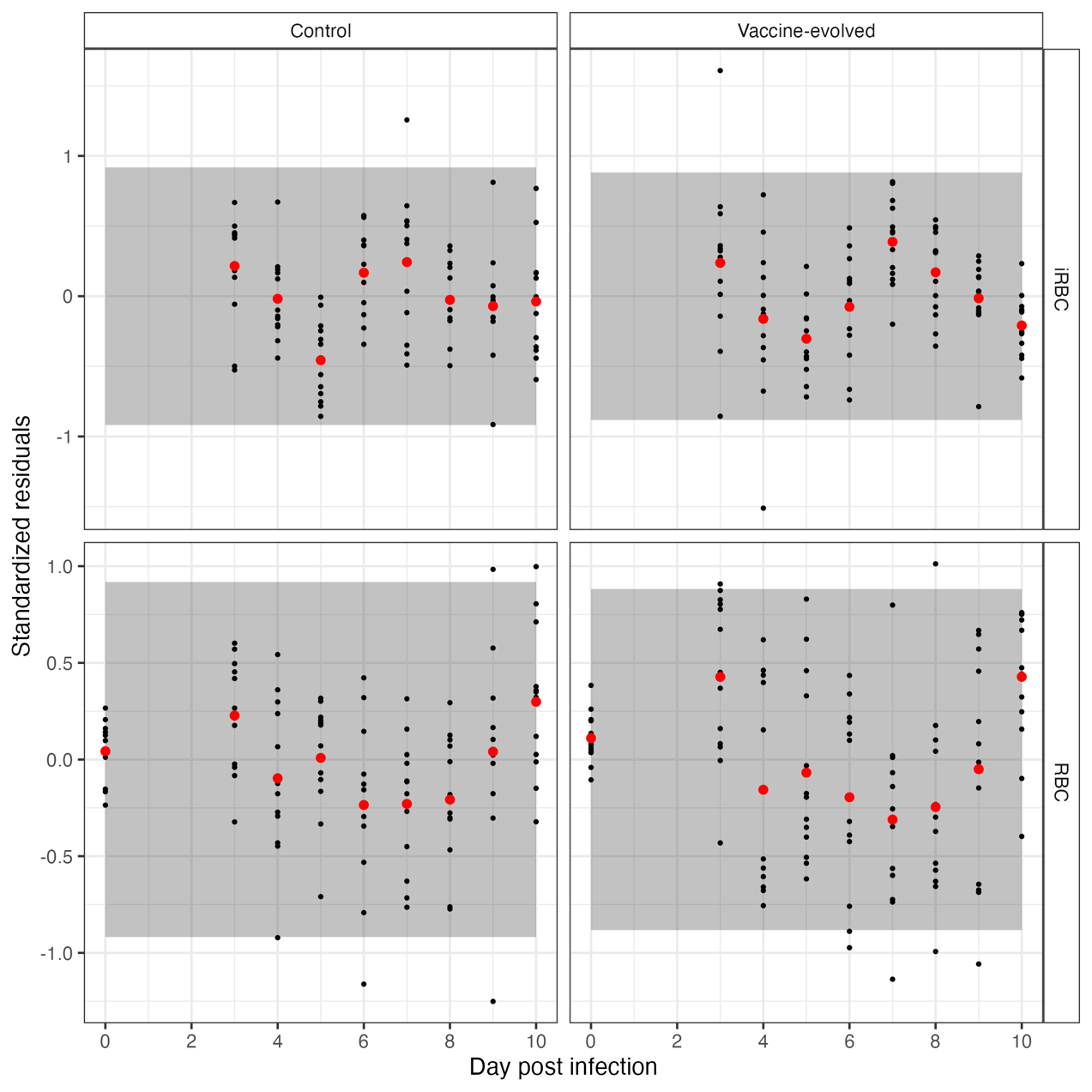


Figure S 1. Standardized residuals for the two treatments (columns) for each data type (rows). The grey bands are Bonferroni-corrected 95% confidence intervals, which are the same across all days for each treatment as there are the same number of data points for each day within each treatment. The large, red points are the mean residuals. The mean residuals falling outside of the CI indicate systematic over/underestimation by the model, but all of them lie within the CI.


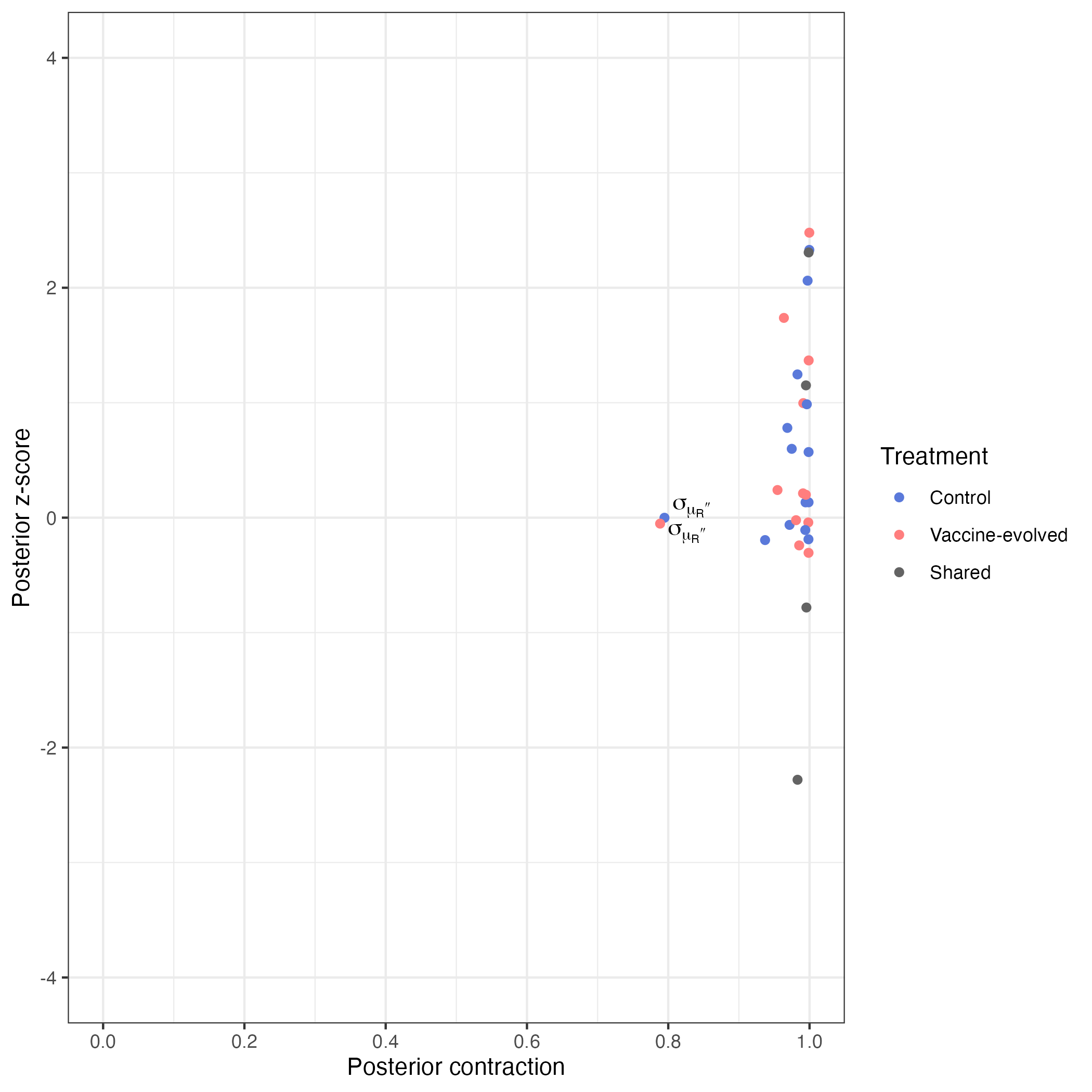


Figure S2. Posterior z-scores (y-axis) and posterior contraction (x-axis). The absolute value of z-scores for all parameters is less than 3, indicating that there are no biases in the parameter estimation process. The posterior contraction is above .9 for all parameters except the two labelled in the plot (representing the standard deviation of the individual-level variation in $\mu_{R}^{''}$ for the two treatments). This indicates that the data are sufficiently informative for estimating the parameters except for those two. We note that the mean value of $\mu_{R}^{''}$ was fixed in the parameter estimating process (fixed value in Table 1) and so we interpret this plot as telling us that the data are not informative for understanding individual variation in density-independent background RBC production.


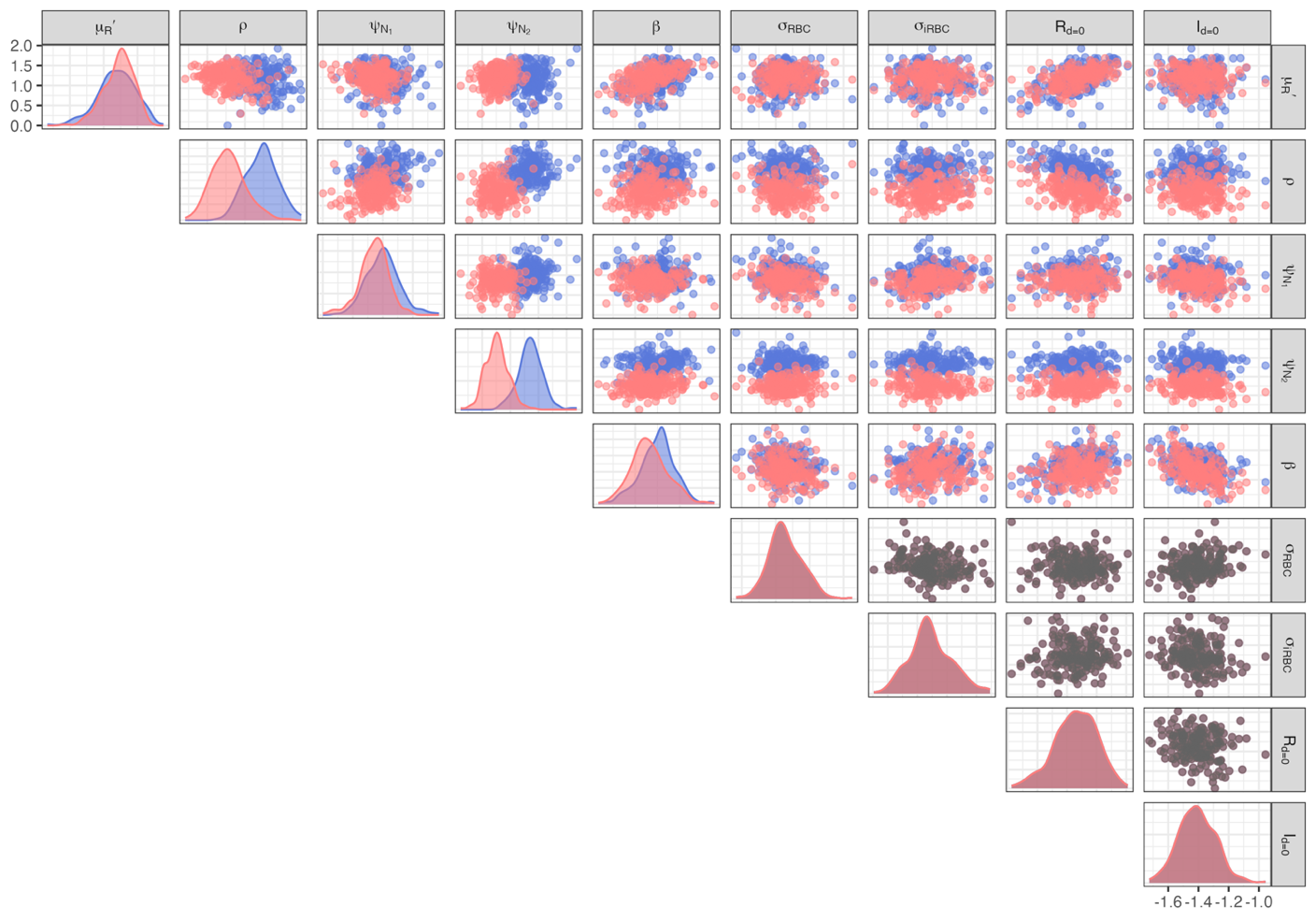


Figure S3 Correlations between treatment mean parameter estimates within each treatment ($\mu_{R}^{'},\psi_{N1}, \psi_{N2}, \rho, \beta$) or with mean parameter values that are shared between treatments ($\sigma_{RBC}$, $\sigma_{iRBC}$, $R_{\left( d=0 \right)}$, $I_{d=0}$). Posterior samples from the vaccine-evolved and the control treatment are shown in pink and blue, respectively. All correlation coefficients are below 0.7 (the highest correlation coefficient is 0.595, between control treatment $\mu_{R}^{'}$ and $R_{\left( d=0 \right)}$), indicating no issues with parameter identifiability.


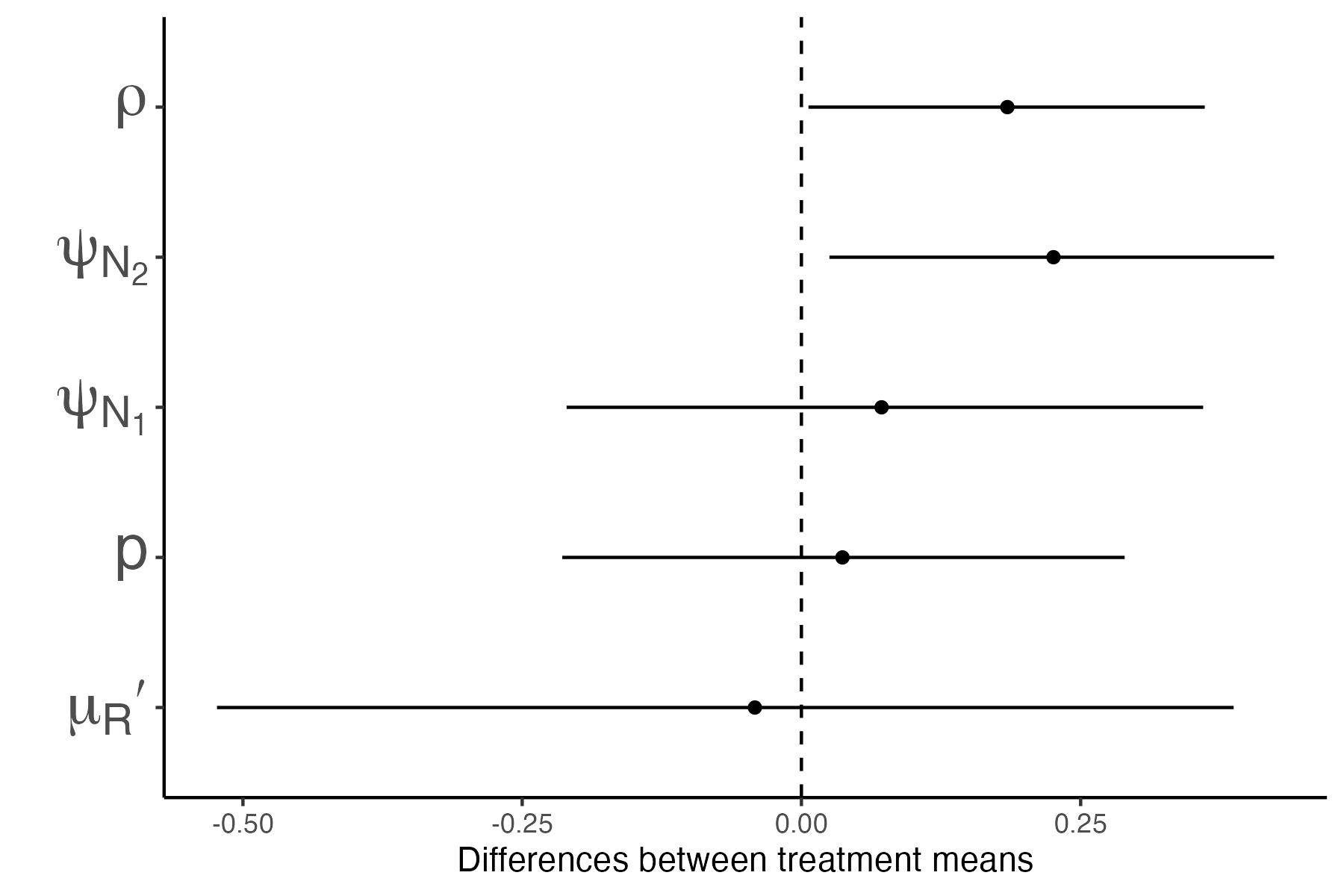


Figure S4 Forest plot of differences between the estimated treatment means when invasion rate ($p$) is fitted and the burst size ($\beta$) is fixed. As with Fig. 5, The horizontal lines indicate 95% credible intervals and the points indicate the means. Deviations of the lines from being centered at zero indicate a difference between the treatments. As in the main text (Fig. 5), targeted immune killing, $\psi_{N_{2}}$, and the density-dependent rate of RBC production, $\rho$, are lower in infections with vaccine-evolved treatment parasites.

Table S1 Table of correlations between each model parameter and first two PC axes from (Figure 6). To assess the alignment of the model parameters with the principal component axes (from Figure 6 in the main text), we include a table of correlations

| Model parameter | PC1 | PC2 |
| --- | --- | --- |
| $\mu_{R}^{'}$ | -0.645 | 0.258 |
| $\rho$ | -0.816 | 0.164 |
| $\psi_{N1}$ | -0.087 | 0.939 |
| $\psi_{N2}$ | -0.776 | 0.0614 |
| $\beta$ | -0.733 | -0.456 |
